# Predicting the Success of Catheter Drainage in Infected Necrotising Pancreatitis: A Cross-Sectional Observational Study

**DOI:** 10.7759/cureus.32289

**Published:** 2022-12-07

**Authors:** Gowtham Sundaram Venkatesan, Srivishnu Thulasiraman, Balaji Kesavan, Dharshana Saravanan, Nithyapriya Chinnaraju

**Affiliations:** 1 Colorectal Surgery, James Cook University Hospital, Middlesbrough, GBR; 2 Upper Gastrointestinal Surgery, James Cook University Hospital, Middlesbrough, GBR; 3 Anaesthesiology, University Hospital of North Tees, Stockton-on-Tees, GBR; 4 General Medicine, James Cook University Hospital, Middlesborough, GBR; 5 Ophthalmology, KMCH (Kovai Medical Center & Hospital) Institute of Health Sciences And Research, Coimbatore, IND

**Keywords:** bisap score, predictive factor, severe acute pancreatitis, percutaneous catheter drainage, severe pancreatitis, necrotising pancreatitis

## Abstract

Background

Management of acute necrotising pancreatitis is often challenging for clinicians. Secondary infection of the necrotic collections leads to sepsis and warrants intervention. Minimally invasive techniques like catheter drainage have recently been proposed over more risky and morbid traditional open procedures. Factors that can predict successful catheter drainage of the necrotic pancreatic collection are still unclear and not well established.

Materials and methods

This study is designed as a retrospective cross-sectional observational study to investigate the association of 21 factors in predicting successful catheter drainage. Data from 30 patients admitted with acute necrotising pancreatitis treated with catheter drainage were collected and analysed. Twenty-one factors, including demographic variables, disease severity factors, drainage criteria, and morphological criteria on imaging, were studied for their predictive association with successful outcomes. Univariate analysis was done for each variable against the outcome. The study was conducted between December 2012 to March 2017. P-value <0.05 was considered statistically significant.

Results

Patients with no organ involvement responded better to primary catheter drainage. Patients with BMI>25 and multi-organ failure were poor candidates for primary catheter drainage. Clinically unwell patients with a Bedside Index for Severity in Acute Pancreatitis (BISAP) score of ≥4 had a negative outcome on catheter drainage and usually ended up in a surgical procedure or eventually succumbed to the disease. Other variables included in our study did not statistically associate with the success or failure of percutaneous catheter drainage.

Conclusion

BMI >25, multiple organ failure, and BISAP score ≥ 4 are independent negative predictors for the success of catheter drainage in infected necrotising pancreatitis. No organ failure showed a positive predictor for successful catheter drainage. Further studies are required to explore these predictive factors in a larger sample size to predict the success of catheter drainage in infected pancreatic necrosis.

## Introduction

One-third of patients with acute necrotising pancreatitis develop secondary infection of necrosis, which often leads to sepsis, multiple organ failure, and death [1,2]. Most patients with infected necrotising pancreatitis may need invasive intervention [3]. However, the traditional approach has long been primary open necrosectomy [2,4]. Recent guidelines advise a step-up approach as an initial invasive treatment [5]. The step-up approach consists of primary catheter drainage followed by minimally invasive necrosectomy if the first modality fails to improve the clinical condition [6]. Catheter drainage is most often performed percutaneously under the guidance of computed tomography (CT) or transabdominal ultrasound [7,8] but can also be performed endoscopically [9,10].

The rationale behind catheter drainage is to control the infection source by removing infected fluid from the necrotic collection [7,11]. Catheter drainage may result in clinical improvement and allow further encapsulation of necrotic collections. The drain technically facilitates necrosectomy at a later stage, reducing the risk of complications such as bleeding and visceral injury [12,13]. Catheter drainage often wholly obviates the need for surgical necrosectomy in at least one-third of patients [8,14]. Moreover, if additional necrosectomy is needed, the retroperitoneal percutaneous catheter can be used as a guide for minimally invasive retroperitoneal necrosectomy [15,16]. Clinical disease severity, size, location, and distribution of peripancreatic collections on CT imaging varies widely in infected necrotising pancreatitis. It is currently unknown which subgroup of patients can be successfully treated with catheter drainage alone and which patients will require additional necrosectomy.

Objective predictors for any invasive procedure will guide clinical decisions and prognostication. Predicting the success of catheter drainage is clinically relevant because the timing of stepping up may be altered based on this prediction. In patients with a high chance of success with catheter drainage, prolonged treatment without necrosectomy could be justified. Conversely, a necrosectomy can be planned shortly after catheter drainage if the patient fails to improve clinically for patients with poor success chances of catheter drainage. Therefore, the prediction of factors for the success of catheter drainage contributes to guiding clinical decisions and counselling patients during the disease course [7,14].

In addition, factors predicting catheter drainage can be used for severity stratification in clinical studies on intervention. However, there is scanty literature that reveals these factors. This study aims to evaluate the factors that have a predictive role in the successful outcome of catheter drainage of infected pancreatic necrosis [8,11].

## Materials and methods

This study was conducted in the tertiary-care hospital Kovai Medical Centre and Hospital, Coimbatore, India. Approval from the ethics committee and scientific research committee of Kovai Medical Centre and Hospital (Approval number: EC/AP/491/12/2016) was obtained before conducting the study.

The study was designed to be a cross-sectional study. The retrospective data were obtained from the medical records of 30 patients treated by catheter drainage for infected pancreatic necrosis following acute pancreatitis. No intervention was done for study purposes. Acute severe pancreatitis is a disease with high morbidity and mortality. Even though there is a variety of treatment modalities, only a subset of patients was treated with catheter drainage. This study's inclusion timeframe was almost four years, from December 2012 to March 2017.

Patients with CT-confirmed infected pancreatic necrosis requiring drain insertion either percutaneously or endoscopically were included. Patients with chronic pancreatitis, alternative septic sources, and collections in the body were excluded. Patients treated by surgical drainage for initial management were also excluded. Validating the retrospective data is essential to determine population size. Due to a smaller number of focus group patients admitted in a year, the study period was planned from 2012 -2017. The following equation is used to estimate the sample size (n)

$$n=N\times \frac{X}{(X+N-1)} ; X=\frac{Z_{\alpha /2}^{2} \times p \times (1-P)}{MOE^{2}}$$

Z α/2 is the critical value of the normal distribution at α/2 (e.g., for a confidence level (CI) of 95%, α is 0.05, and the critical value is 1.96), MOE is the margin of error, p is the sample proportion, and N is the population size. Note that a finite population correction has been applied to the sample size formula. The values given to compute the sample size are as follows: MOE=5%, CI=95%, population = 42, and sample proportion = 90%. Therefore, the sample size came to around 31. Table 1 lists all predictive factors that were used in our study. There were 18 factors analysed under four major categories: demographic factors, drainage factors, disease security variables (<24 hours), and morphological variables based on CT findings.

**Table 1:** Predictive factors BMI: body mass index; BISAP: Bedside Index for Severity in Acute Pancreatitis; CTSI: CT severity index

Demographic factors	Disease severity variables (<24 hours)
Age, Sex, BMI, Aetiology, Co-morbidities (Diabetes)	BISAP score, Serum leukocytes, Single/multiple organ failure
Drainage factors	Morphological variables on CT
Days from admission to primary catheter drainage, Size of primary catheter drain, Separate drain for separate collections, Upsizing primary catheter drain to larger drain Largest catheter drain	Pancreatic necrosis or extrapancreatic necrosis only percentage of pancreatic necrosis, CTSI score, Pattern of pancreatic necrosis, Size of the collection, Spread of the collection, Degree of encapsulation of the collection, Contents of the collection

IBM SPSS Statistics for Windows, Version 20.0 (Released 2011; IBM Corp., Armonk, New York, United States) was employed to analyse the data. Continuous data were analysed for its mean, median and standard deviation. The categorical variable was analysed using the chi-square test. Univariate analysis of the variables for their association in predicting the success of the catheter drainage was done individually for each predictor. Pearson Chi-square value was calculated for the individual predictors. The percentage of the values within the results and the group have been described separately. Though these were not statistically important, the data were analysed, and outcomes were interpreted with appropriate discussion based on the clinical significance. Chi-square analysis was performed for statistical significance. The p-value was calculated for individual variables. A result was deemed statistically significant when the p-value was ≤ 0.05

## Results

Data from 30 patients were collected and formulated into tables for straightforward interpretation. We obtained the following results by analysing the data (Table 2). The descriptive data of the values in the following tables. In the study population, the mean participant age was 43 ± 14 years, with more than three-fourths being male, 86.7% (26/30), and 13.3% being females. The mean duration of stay with acute severe necrotising pancreatitis was 28 ± 15 days. Out of 30 patients, 26 required early catheter drainage of the infected pancreatic collections, i.e., within 14 days of presentation.

**Table 2:** Factors analysed with regard to the success of catheter drainage SD: standard deviation; BISAP: Bedside Index for Severity in Acute Pancreatitis; WBC: white blood cell count; PN: pancreatic necrosis; EPN: extra-pancreatic necrosis; CTSI: CT severity index

FACTORS	OUTCOME
Demographics	
Age (mean ± S.D)	42.6 (13.73)
Sex, no (%)	
Male	26 (86.67)
Female	4 (13.33)
Duration of in days (mean± S.D)	28.17± 15.36
BMI, no (%)	
<25	22 (73.33)
>=25	8 (26.6)
Associated diabetes, no (%)	7 (23.3)
Days since admission to catheter drainage, no (%)	
Early (<14days)	25 (83.33)
Late (>= 14days)	5 (16.67)
Drainage Criteria	
Separate drain for separate collections, no (%)	8 (26.67)
Upsizing of catheter, no (%)	8 (26.67)
Largest catheter used, no (%) stay	
<=10	4 (13.33)
>10, <18	22 (73.33)
>=20	4 (13.33)
Disease Severity	
BISAP score, no (%)	
1	1 (3.33)
2	5 (16.67)
3	13 (43.33)
4	11 (36.67)
Organ failure, no (%)	
No organ failure	10 (33.3)
Single organ failure	5 (16.67)
Multiple organ failure	15 (50)
Total WBC counts, no (%)	
<4000	2 (6.67)
4000-11000	2 (6.67)
11000-20000	14 (46.67)
>20000	12 (40)
Morphological Criteria	
Percentage of pancreatic necrosis, no (%)	
<30	9 (30)
>30	18 (60)
>50	2 (6.67)
>70	1 (3.33)
CTSI (no (%)	
Mild	1 (3.33)
Severe	29 (96.67)
Pattern of necrosis, no (%)	
Central	11 (36.67)
Left	3 (10)
Right	5 (16.67)
Scattered	1 (3.33)
Subtotal	10 (33.33)
Pancreatic PN/extra-pancreatic necrosis EPN, no (%)	
PN and EPN	17 (56.67)
PN	12 (40)
EPN	1 (3.33)
Degree of encapsulation, no (%)	
Some wall formation	7 (23.3)
Complete wall formation	5 (16.67)
No visible wall formation	18 (60)
Content of collection, no (%)	
Heterogenous	13 (43.33)
Heterogenous + gas	1 (3.33)
Homogenous + gas	1 (3.33)
Homogenous	5 (16.67)
Impacted gas bubbles	10 (33.33)
Spread of collection	
Bilateral	5 (16.67)
Central	10 (33.33)
Left	9 (30.00)
Pelvis	1 (3.33)
Right	5 (16.67)
Size of collection in ml (mean ± S.D)	809.67± 530.11
Endpoints	
Re-admission	13 (43.33%)
Necrosectomy	10 (33.33%)
Mortality	7 (23.3%)
Success of catheter drainage	16 (53.33%)

Sixty-nine percent (21/30) of the cases had more than 30% of pancreatic necrosis, most of which showed 30-50% necrosis. More than 95% of the patients were labelled as having severe pancreatitis according to the CTSI. The heterogeneous collection was observed in 13% of the cases. The most frequent site of spread of collection was in the central (33%), followed by the left paracolic gutter, which was 9%. The mean size of the collection was 810ml ±530ml.

The success of catheter drainage was 53% (16/30) in the study population. Despite minimally invasive catheter drainage, 33% (10/30) of the cases underwent necrosectomy. Forty-three percent (13/30) of the patients needed re-admission while managing infected pancreatic necrosis. The mortality rate of infective pancreatic necrosis was reported as 23.3%.

Univariate analysis of the variables and their association in predicting the success of the catheter drainage was done individually for predictors. Table 3 shows gender distribution and its relation to the success of catheter drainage. Even though there is a significantly higher proportion of males (26/30) to females (4/30), the analysis did not reveal significant differences among them concerning the success of the catheter drainage (p=0.222)

**Table 3:** Gender and its relation to the success of catheter drainage Pearson Chi-square value 1.489; df = 1; p-value 0.222

Sex		Results Status		Total
		Success	Failure	
Male	Count	15	11	26
	% Within Sex	57.7%	42.3%	100.0%
	% Within Results	93.8%	78.6%	86.7%
Female	Count	1	3	4
	% Within Sex	25.0%	75.0%	100.0%
	% Within Results	6.2%	21.4%	13.3%
Total	Count	16	14	30
	% Within Sex	53.3%	46.7%	100.0%
	% Within Results	100.0%	100.0%	100.0%

Table 4 shows age distribution and its relation to the success of catheter drainage. The analysis did not reveal significant differences among different age groups regarding the success of catheter drainage (p=0.946).

**Table 4:** Age distribution and its relation to the success of catheter drainage Pearson Chi-square value 0.111; df = 2; p-value 0.946

Age		Results Status		Total
		Success	Failure	
<31 years	Count	3	2	5
	% Within Age	60.00%	40.00%	100.00%
	% Within Results Status	18.80%	14.30%	16.70%
31 - 60 years	Count	12	11	23
	% Within Age	52.20%	47.80%	100.00%
	% Within Results Status	75.00%	78.60%	76.70%
>60 years	Count	1	1	2
	% Within Age	50.00%	50.00%	100.00%
	% Within Results Status	6.20%	7.10%	6.70%
Total	Count	16	14	30
	% Within Age	53.30%	46.70%	100.00%
	% Within Results Status	100.00%	100.00%	100.00%

Most participants (73.3%, 22/30) had a BMI of more than 25, whereas only around a quarter percentage (26.6%, 8/30) had average/low BMI. Table 5 shows the relationship between BMI and the success of catheter drainage. The statistical analysis revealed that a BMI<25 was significantly associated with the success of catheter drainage, whereas BMI >25 is associated with failure (p=0.007).

**Table 5:** BMI and its relation to the success of catheter drainage Pearson Chi-square value 7.308; df = 1; p-value 0.007

BMI		Results Status		Total
		Success	Failure	
<25	Count	15	7	22
	% Within BMI	68.2%	31.8%	100.0%
	% Within Results	93.8%	50.0%	73.3%
>= 25	Count	1	7	8
	% Within BMI	12.5%	87.5%	100.0%
	% Within Results	6.2%	50.0%	26.7%
Total	Count	16	14	30
	% Within BMI	53.3%	46.7%	100.0%
	% Within Results	100.0%	100.0%	100.0%

Various etiological factors (Alcohol, biliary, drug-induced, unknown) were analysed for their association with the success of catheter drainage (Table 6), and the analysis did not reveal any statistical significance among the etiological factor (p=0.104).

**Table 6:** Aetiology of pancreatitis and its relation to the success of catheter drainage Pearson Chi-square value 6.161; df = 3; p-value 0.104

Aetiology		Results Status		Total
		Success	Failure	
Alcoholic	Count	5	10	15
	% Within Aetiology	33.3%	66.7%	100.0%
	% Within Results	31.2%	71.4%	50.0%
Biliary	Count	8	2	10
	% Within Aetiology	80.0%	20.0%	100.0%
	% Within Results	50.0%	14.3%	33.3%
Drug-Induced	Count	1	0	1
	% Within Aetiology	100.0%	.0%	100.0%
	% Within Results	6.2%	.0%	3.3%
Unknown	Count	2	2	4
	% Within Aetiology	50.0%	50.0%	100.0%
	% Within Results	12.5%	14.3%	13.3%
Total	Count	16	14	30
	% Within Aetiology	53.3%	46.7%	100.0%
	% Within Results	100.0%	100.0%	100.0%

Diabetes was associated with co-morbidity in only seven participants (7%). Table 7 shows diabetes and its relation to the success of catheter drainage. Analysis showed that more than 75% (23/30) of the study population were non-diabetic. Even though catheter drainage was successful in 60.9% (14/23) of the non-diabetic population, no statistical significance was established (p=0.134)

**Table 7:** Diabetes and its relation to the success of catheter drainage Pearson Chi-square value 2.249; df = 1; p-value 0.134

Diabetes		Results Status		Total
		Success	Failure	
Yes	Count	2	5	7
	% Within Diabetes	28.6%	71.4%	100.0%
	% Within Results	12.5%	35.7%	23.3%
No	Count	14	9	23
	% Within Diabetes	60.9%	39.1%	100.0%
	% Within Results	87.5%	64.3%	76.7%
Total	Count	16	14	30
	% Within Diabetes	53.3%	46.7%	100.0%
	% Within Results	100.0%	100.0%	100.0%

Table 8 shows early (<14 days) and delayed (≥14 days) catheter drainage and its relation to the success of the treatment. It shows that most patients underwent early drainage (83.3%, 25/30). The analysis did not reveal statistical significance between the two groups (p=0.513).

**Table 8:** Time of catheter drainage and its relation to the success Pearson Chi-square value 0.429; df = 1; p-value 0.513

Time of Catheter Drainage		Results Status		Total
		Success	Failure	
<14 days	Count	14	11	25
	% Within group	56.0%	44.0%	100.0%
	% Within Results	87.5%	78.6%	83.3%
>= 14 days	Count	2	3	5
	% Within group	40.0%	60.0%	100.0%
	% Within Results	12.5%	21.4%	16.7%
Total	Count	16	14	30
	% Within group	53.3%	46.7%	100.0%
	% Within Results	100.0%	100.0%	100.0%

In eight cases, separate drains were used for multiple isolated intra-abdominal collections. Table 9 depicts the relation between the success of catheter drainage and each separate catheter for independent display. The analysis found no statistically significant differences between the two groups (p=0.825).

**Table 9:** Strategy of a separate catheter for separate collection and its relation to the success of catheter drainage Pearson Chi-square value 0.049; df = 1; p-value 0.825

Separate Catheter for Separate Collections		Results Status		Total
		Success	Failure	
Yes	Count	4	4	8
	% Within group	50.0%	50.0%	100.0%
	% Within Results	25.0%	28.6%	26.7%
No	Count	12	10	22
	% Within group	54.5%	45.5%	100.0%
	% Within Results	75.0%	71.4%	73.3%
Total	Count	16	14	30
	% Within group	53.3%	46.7%	100.0%
	% Within Results	100.0%	100.0%	100.0%

On analysing the drainage criteria, 26.7% (8/30) of the participants had upsizing pigtail catheters due to various reasons such as blockage and sepsis. Table 10 shows the relation between upsizing the catheter and the success of catheter drainage. Eight out of 30 underwent upsizing of the catheter to aid in adequate draining of collection and control of sepsis. The analysis did not reveal the significance of upsizing the catheter (p= 0.061).

**Table 10:** Strategy of catheter upsizing and its relation to the success of catheter drainage Pearson Chi-square value 3.519; df = 1; p-value 0.061

Catheter Upsizing		Results Status		Total
		Success	Failure	
Yes	Count	2	6	8
	% Within group	25.0%	75.0%	100.0%
	% Within Results	12.5%	42.9%	26.7%
No	Count	14	8	22
	% Within group	63.6%	36.4%	100.0%
	% Within Results	87.5%	57.1%	73.3%
Total	Count	16	14	30
	% Within group	53.3%	46.7%	100.0%
	% Within Results	100.0%	100.0%	100.0%

Various sizes of pigtail catheters were used based on the type of collection and the availability of the catheter. A range of catheters between 10 and 18 French was used in large proportion in 73% (22/30) of the cases. Table 11 shows the relation between the size of the catheter and the success of catheter drainage. Catheter were categorised into three groups (<10F,11-20F,>20F) based on their lumen size. There was no significant difference in the success of catheter draining among the groups (p=0.172).

**Table 11:** Catheter size and its relation to the success of catheter drainage Pearson Chi-square value 3.519; df = 2; p-value 0.172

Catheter Size		Results Status		Total
		Success	Failure	
Small (<=10)	Count	1	3	4
	% Within group	25.0%	75.0%	100.0%
	% Within Results	6.2%	21.4%	13.3%
Moderate (11 - 20)	Count	14	8	22
	% Within group	63.6%	36.4%	100.0%
	% Within Results	87.5%	57.1%	73.3%
Large (>20)	Count	1	3	4
	% Within group	25.0%	75.0%	100.0%
	% Within Results	6.2%	21.4%	13.3%
Total	Count	16	14	30
	% Within group	53.3%	46.7%	100.0%
	% Within Results	100.0%	100.0%	100.0%

BISAP score, the most straightforward tool to calculate the mortality risk, showed that nearly 80% of the participants had a score of ≥ 3. Table 12 shows the BISAP score and its relation to the success of catheter drainage. When the BISAP score increased from 1 to 3, success rate gradually decreased. Notably, the failure rate reached a peak of 91% when the BISAP score was 4. In addition, the analysis showed a significant association between the BISAP score and the outcome following catheter drainage (p=0.003).

**Table 12:** BISAP score and its relation to the success of catheter drainage BISAP: Bedside Index for Severity in Acute Pancreatitis Pearson Chi-square value 13.861; df = 3; p-value 0.003

BISAP Score		Results Status		Total
		Success	Failure	
1	Count	1	0	1
	% Within BISAP score	100.0%	.0%	100.0%
	% Within Results	6.2%	.0%	3.3%
2	Count	4	1	5
	% Within BISAP score	80.0%	20.0%	100.0%
	% Within Results	25.0%	7.1%	16.7%
3	Count	10	3	13
	% Within BISAP score	76.9%	23.1%	100.0%
	% Within Results	62.5%	21.4%	43.3%
4	Count	1	10	11
	% Within BISAP score	9.1%	90.9%	100.0%
	% Within Results	6.2%	71.4%	36.7%
Total	Count	16	14	30
	% Within BISAP score	53.3%	46.7%	100.0%
	% Within Results	100.0%	100.0%	100.0%

Organ failure is one of the recognised complications of acute pancreatitis. Tables 13, 14, 15 show the relationship between the number of organ failures and the success of catheter drainage. Patients without organ failure (10/30) showed a significant 100% success rate following catheter drainage (p<0.05). While 10 out of 30 patients had a multi-organ failure, there was consistently a substantial failure rate of 66.7%, and the findings are statistically significant (p=0.028). The single organ failure group failed to show a significant association with the success of the treatment (p=0.102).

**Table 13:** No organ failure and its relation to the success of catheter drainage Pearson Chi-square value 13.125; df = 1; p-value < 0.05

No Organ Failure		Results Status		Total
		Success	Failure	
Yes	Count	10	0	10
	% Within no organ failure	100.0%	.0%	100.0%
	% Within results status	62.5%	.0%	33.3%
No	count	6	14	20
	% Within no organ failure	30.0%	70.0%	100.0%
	% Within results status	37.5%	100.0%	66.7%
Total	count	16	14	30
	% Within no organ failure	53.3%	46.7%	100.0%
	% Within results status	100.0%	100.0%	100.0%
Pearson Chi-square value 13.125; df = 1; p-value < 0.05				

**Table 14:** Single organ failure and its relation to the success of catheter drainage Pearson Chi-square value 2.679; df = 1;  p-value = 0.102

Single Organ Failure		Results Status		Total
		Success	Failure	
Yes	Count	1	4	5
	% Within single organ failure	20.0%	80.0%	100.0%
	% Within results	6.2%	28.6%	16.7%
No	count	15	10	25
	% Within single organ failure	60.0%	40.0%	100.0%
	% Within results	93.8%	71.4%	83.3%
Total	count	16	14	30
	% Within single organ failure	53.3%	46.7%	100.0%
	% Within Results	100.0%	100.0%	100.0%

**Table 15:** Multi-organ failure and its relation to the success of catheter drainage Pearson Chi-square value 4.821; df = 1;  p-value =0.028

Multiple Organ Failure		Results Status		Total
		Success	Failure	
Yes	Count	5	10	15
	% within multiple organ failure	33.3%	66.7%	100.0%
	% within results status	31.2%	71.4%	50.0%
No	count	11	4	15
	% within multiple organ failure	73.3%	26.7%	100.0%
	% within results status	68.8%	28.6%	50.0%
Total	count	16	14	30
	% within multiple organ failure	53.3%	46.7%	100.0%
	% within Results Status	100.0%	100.0%	100.0%

White cell count (WCC) was analysed on the day of admission, which showed 46.7% (14/30) had WCC between 11-20,000 and 12% had significantly raised WCC of more than 20,000. Table 16 illustrates the relation between WCC and the success of catheter drainage. Based on cell counts, the cases were categorised into four groups: 4000, 4000-11000, 11-20,000, and >20,000. Analysis shows no significant difference among groups with the success of catheter drainage (p= 0.977).

**Table 16:** Total leucocyte count and its relation to the success of catheter drainage Pearson Chi-square value 0.201; df = 3;  p-value =0.977

Total Count		Results Status		Total
		Success	Failure	
<4000	Count	1	1	2
	% within TC	50.0%	50.0%	100.0%
	% within Result	6.2%	7.1%	6.7%
4000 - 11000	Count	1	1	2
	% within TC	50.0%	50.0%	100.0%
	% within Results	6.2%	7.1%	6.7%
11000 - 20000	Count	7	7	14
	% within TC	50.0%	50.0%	100.0%
	% within Results	43.8%	50.0%	46.7%
>20000	Count	7	5	12
	% within TC	58.3%	41.7%	100.0%
	% within Results	43.8%	35.7%	40.0%
Total	Count	16	14	30
	% within TC	53.3%	46.7%	100.0%
	% within Results	100.0%	100.0%	100.0%

The relation between pancreatic necrosis and the success of catheter drainage is analysed in Table 17. Based on CT imaging results, necrosis was classified into three categories: pancreatic necrosis with extrapancreatic necrosis, pancreatic necrosis alone, and extrapancreatic necrosis alone. The analysis does not reveal a significant impact on the outcome of catheter drainage among the groups (p=0.628).

**Table 17:** Pancreatic/extra-pancreatic necrosis and its relation to the success of catheter drainage Pearson Chi-square value 0.930; df = 2;  p-value 0.628

Pancreatic Necrosis		Results Status		Total
		Success	Failure	
Pancreatic and extra-pancreatic necrosis	Count	9	8	17
	% within Pancreatic necrosis	52.9%	47.1%	100.0%
	% within Results Status	56.2%	57.1%	56.7%
pancreatic necrosis	Count	6	6	12
	% within Pancreatic necrosis	50.0%	50.0%	100.0%
	% within Results Status	37.5%	42.9%	40.0%
extra-pancreatic necrosis	Count	1	0	1
	% within Pancreatic necrosis	100.0%	.0%	100.0%
	% within Results Status	6.2%	.0%	3.3%
Total	Count	16	14	30
	% within Pancreatic necrosis	53.3%	46.7%	100.0%
	% within Results Status	100.0%	100.0%	100.0%

Table 18 shows the quantity of pancreatic necrosis and its relation to the success of catheter drainage. Pancreatic necrosis is divided into two groups with a cut-off of 30%, and analysis showed no significant difference between the level of necrosis about the outcome of catheter drainage (p=0.523). 

**Table 18:** Percentage of pancreatic necrosis and its relation to the success of catheter drainage

Percentage of Pancreatic Necrosis		Results Status		Total
		Success	Failure	
<30	Count	4	5	9
	% within Pancreatic necrosis %	44.4%	55.6%	100.0%
	% within Results Status	25.0%	35.7%	30.0%
>=30	Count	12	9	21
	% within Pancreatic necrosis %	57.1%	42.9%	100.0%
	% within Results Status	75.0%	64.3%	70.0%
Total	Count	16	14	30
	% within Pancreatic necrosis %	53.3%	46.7%	100.0%
	% within Results Status	100.0%	100.0%	100.0%
Pearson Chi-square value 0.408; df = 1; p-value 0.523				

Table 19 shows the relation of the CTSI score with the success of the catheter draining. Of 30 pateints, 29 presented with severe pancreatitis according to CTSI scoring. Out of 29 people within the extreme group, success rate was equivocal, and analysis showed no significant difference between the two groups regarding the outcome of catheter drainage (p=0.341).

**Table 19:** CTSI score and its relation to the success of catheter drainage CTSI: CT severity index

CTSI		Results Status		Total
		Success	Failure	
Mild (<=6)	Count	1	0	1
	% within CTSI	100.0%	.0%	100.0%
	% within Results	6.2%	.0%	3.3%
Severe (>6)	Count	15	14	29
	% within CTSI	51.7%	48.3%	100.0%
	% within Results	93.8%	100.0%	96.7%
Total	Count	16	14	30
	% within CTSI	53.3%	46.7%	100.0%
	% within Results	100.0%	100.0%	100.0%
Pearson Chi-square value 0.905; df = 1; p-value 0.341				

## Discussion

This observational study was done with 30 patients diagnosed with acute necrotising pancreatitis. Infected necrotising pancreatitis was mainly diagnosed with abdominal contrast CT, clinical findings, and laboratory parameters. Clinical criteria here included pancreatitis with worsening clinical features like high-grade temperature, tachycardia, tachypnoea, hypoxia, respiratory distress, hypotension, and dropping urine output. But this has not been used as a sole criterion for diagnosis of infection. This was supplemented by the CT scan, cultures, and other lab tests. Data, including demographics, disease severity, drainage details, and morphological criteria, are listed in Table 2. The catheter drainage was performed for infected pancreatic necrosis, peripancreatic necrosis, or both. In this study, all 30 patients underwent image-guided percutaneous catheter drainage as a drain tube or a pigtail catheter.

The outcome of the catheter drainage was considered a failure if it required any form of surgical intervention or death. Surgical intervention options for necrotising pancreatitis included but were not limited to video-assisted retroperitoneal necrosectomy, minimally invasive retroperitoneal necrosectomy, and open necrosectomy. Our study defines successful catheter drainage as survival without needing surgical or endoscopic necrosectomy.

Out of 30 patients recruited in the study, 16 had successful catheter drainage, four worsened and died despite catheter drainage, and 10 required further necrosectomy by stepwise approach. Three of the 10 patients who required necrosectomy died eventually, despite catheter drainage and necrosectomy. All three patients who died had worsening multiple organ failure, unstable hemodynamic profiles, unfavourable BISAP scores, and laboratory investigations.

In our study, the success rate of catheter drainage was 16/30 (53.3 %), which was comparable to the success rates of other studies [7,17]. A multicentre randomised control trial compared the stepwise surgical approach to the traditional open necrosectomy, showing a success rate of 35% [18-20]. Several factors influence the success of catheter drainage in necrotising pancreatitis.

Recently, the stepwise approach in managing acute necrotising pancreatitis has become more popular, replacing traditional open necrosectomy, as the latter has a mortality rate of 7-39% [21-23]. It involves image-guided (USG/CT guided) drainage of necrotic collection to control sepsis. If it fails, minimally invasive procedures such as endoscopic guided transgastric necrosectomy or retroperitoneal video-assisted laparoscopy/rigid nephroscopy/endoscopy are performed. Finally, if all those mentioned above do not work out, it can proceed with a minimally invasive open necrosectomy [14]. The primary rationale behind this approach is to control the sepsis rather than complete the removal of necrotic material and to prevent clinical deterioration of the patient with a morbid invasive procedure. Additionally, this avoids complications such as iatrogenic trauma to normal pancreatic parenchyma and controls the stress response to surgery in critically ill patients. 

Demographic variables

Out of 30 patients, 26 were males and four were females. Out of four females, only one had successful drainage, two underwent a surgical necrosectomy, and the fourth died. Out of 26 males, 15 (57.7%) had successful drainage. However, the statistical analysis failed to prove a significance of the sex of the patient in predicting success, as shown in Table 3. Univariate regression analysis required a minimum of five patients in each subset for significance. Less number of female patients may be a reason for this. In our study, the male gender had a high probability of success (57.7%) compared to the female gender, which is contrary to previous studies, which concluded that the male gender is a negative predictor of catheter drainage in acute necrotising pancreatitis [24,25]. In these studies, the male sex had a higher incidence of sepsis, was more prone to develop multiple organ failure, and required invasive surgical intervention than females.

The mean age of the patients in this study was 42.6 years, with a standard deviation of 13.73. Out of 30 patients, 28 were below 60 years of age. As shown in Table 4, statistical analysis of the age did not have significance as a predictive factor, with a p-value of 0.946 (>0.05).

In our study, out of 30 patients, 22 had BMI < 25. Fifteen of the 22 patients with BMI <25 had successful catheter drainage. This was found to be significantly associated with the success of the drainage with a p-value of 0.007 (p<0.05). The failure rate in the obese group was seven out of eight patients (87.5%). BMI >25 was associated with poor outcomes and failure of catheter drainage (Table 5).

The common etiological factors for acute pancreatitis encountered in this study were alcoholic pancreatitis, biliary, drug-induced, post-endoscopic retrograde cholangiopancreatography (ERCP), and hypercholesterolemia. None of the etiological factors was significantly associated with the successful drainage of the necrotic collection (Table 6). Though not statistically significant, we could derive the following results from our study: Alcohol is the most common etiological factor encountered in this study (15/30). The alcoholic group were more associated with the negative outcome of catheter drainage (66.7%). Biliary pancreatitis had a high probability of success with catheter drainage (8/10, 80%) compared to other factors such as alcohol, drug-induced, idiopathic, and miscellaneous.

The presence of diabetes may complicate the course of the illness. Diabetic patients already have compromised organ functions in micro and macrovascular diseases. However, diabetes as an associated co-morbidity did not show statistical significance in determining the success of catheter drainage (p-value 0.134), as shown in Table 7.

Drainage criteria

The international guideline recommends that the timing of the catheter drainage for necrotising pancreatitis can be postponed up to the stage of walled-off necrosis in severe acute pancreatitis (i.e. four weeks from the onset of acute pancreatitis). However, in our study, early catheter drainage (<14 days) with catheter drainage showed a high probability of success (87.5%) when compared to delayed drainage (12.5%). A recent study comparing the proactive percutaneous drainage strategy (frequent early revision and upsizing of catheter drain) vs standard catheter drainage (maximum 1-2 percutaneous catheter drainage procedures followed by early video-assisted retroperitoneal wound debridement) showed less need for necrosectomy, less incidence of new organ failure, and lower sepsis in the former group [26].

The time since presentation to the hospital and catheter drainage was studied in two groups: early (within 14 days) and late (more than or equal to 14 days). Twenty-five patients underwent early catheter drainage, out of which 14 succeeded (56%). The success rate in the late group was two out of five (40%). This was not statistically significant, as shown in Table 8.

Separate catheters for separate collections were tried in eight patients, which showed a 50% success rate. This was not statistically significant (Table 9). These eight patients had heterogeneous, multi-loculated collections on imaging, which warranted a separate catheter for separate groups to be drained. This cannot be extrapolated to uncomplicated, homogenous, single-cavity low-volume collections. Decisions should be based on a clinical background based on the patient profile, the nature of the group, and the imaging features of that particular patient.

Upsizing the catheter is a strategy for blocked drains, the inability to drain with smaller-size catheters, and collections not decreasing in size in further scans. Nevertheless, this strategy does not usefully improve the outcome of draining the collections, according to the data obtained (Table 10). In our study, the most commonly used modality is CT-guided drainage. The pigtail catheter was used for image-guided drainage, and the drain size varied from 8-28 Fr. Due to the blockage of the drain and inadequate drainage, the drain tube upsizing was done under CT guidance in eight patients. There was no statistical significance noted for separate drain (p<0.061) and upsizing of catheters (p <0.825). Previous studies show no correlation between the drain's number and size to the disease's outcome [14,27,28]. It could be due to the nature of infected pancreatic necrosis, which shows the transition of solid debris to a liquid component, which aids in helps in evacuation of content independent of catheter size.

The size of the catheter drain tube was not a better predictor for successful drainage of pancreatic necrosis, even in previous studies [17]. Our analysis also confirms this data (Table 11). Catheters of sizes 10-28 Fr were used to drain the necrotic collections in our study. Nevertheless, the size of the drain catheter was not statistically associated with the success or failure of catheter drainage.

Disease severity indices

The BISAP score was proposed in 2008 for the early recognition of the risk of in-hospital mortality in acute pancreatitis. This scoring system consists of five variables: blood urea nitrogen (BUN) > 25 mg/dl, impaired mental status, systemic inflammatory response syndrome (SIRS) development, age > 60 years, and presence of pleural effusion. Each variable is given one point, and a cumulative score is calculated. Scores between 0-2 are associated with low mortality of < 2%, while scores ≥ 3 have a higher mortality risk of >5%.

The BISAP score is considered a particular tool in assessing the severity of acute pancreatitis compared to the acute physiology and chronic health evaluation (APACHE) II score and Ranson criteria [29], which showed a strong association with the success of catheter drainage in acute necrotising pancreatitis (p = 0.003). The group population with a BISAP score of 4 had a higher failure rate (90.9%) for catheter drainage. Hence, it can be a valuable predictor of percutaneous catheter drainage outcome (Table 12).

Organ failure is troublesome in acute pancreatitis and it complicates the clinical picture, posing difficulty in managing pancreatic necrosis. Patients with no organ/involvement had an excellent outcome (Table 13). Ten out of 10 patients in the no organ failure group had successful catheter drainage in our study. The p-value was statistically significant (p <0.05) for this factor. This shows that no organ failure is a good predictor of pancreatic catheter drainage and is associated with a favourable outcome. Single organ failure did not predict either way and analysis was not significant as a good or bad predictor (p =0.102) as in Table 14. On the contrary, multiple organ failure suggested a bad outcome in our study (Table 15). Patients with multiple organ failure had a failed catheter drainage and eventually either needed necrosectomy or died during the course of the study. The presence of multiple organ failure shall preclude adopting a catheter drain management (p =0.028).

The onset of multiple organ dysfunction syndrome (MODS) due to SIRS is the most vulnerable entity as a sequela of acute pancreatitis due to the activation of inflammatory cascade via cytokines (tumour necrosis factor (TNF) α and interleukin (IL)-1). In our study, the population with MODS (15/30) was strongly associated with failure of percutaneous catheter drainage (71.4%, p = 0.028). Eventually, the group of people with no single organ failure (10/30) had a robust association with the success of percutaneous catheter drainage (62.5%, p<0.001). Hence, it is considered a valuable negative predictor for catheter drainage in acute necrotising pancreatitis. The mortality among the MODS group is 46.7%, which is par with previous literature, 30% [30] and 45% [14].

Total leucocyte counts were measured at the time of catheter drainage and analysed for their association with the success of percutaneous catheter drainage. Even high leucocyte counts (>20,000) were not associated with failure of catheter drainage (Table 16).

Imaging criteria

Combined pancreatic and extrapancreatic necrosis in the study population was found in 17 patients (56.7%), which is the most common pattern of acute necrotising pancreatitis. Pancreatic necrosis alone was noted in 12 (40%) and extrapancreatic necrosis alone in one patient (3.3%). There was no significant correlation with the prediction of success in catheter drainage outcome observed (p = 0.628). The percentage of pancreatic necrosis as seen in CT scans was analysed in two groups, <30% and >30%. However, this stratification did not give statistically significant information to guide us in predicting a successful outcome of percutaneous catheter drainage (p=0.523) as shown in Table 18.

The CTSI grades the severity of the necrosis in terms of imaging features. Except for one, all patients (n=29) had severe pancreatitis based on CTSI scores (8 and 10). They had nearly equal chances of success or failure following catheter drainage. Fifteen patients had a favourable outcome, and the rest 14 did not. Given equal probability, this is not statistically significant (p=0.341).

The pattern of necrosis (right, left, subtotal, scattered, central), the content of collection (heterogenous, homogenous, impacted gas bubbles), the volume of collections, and the degree of encapsulation all failed to associate or dissociate significantly as a predictor for successful percutaneous catheter drainage.

The common isolates on a microbiological culture were *Acinetobacter*, *Escherichia coli*, *Enterococcus*, *Klebsiella*, *Pseudomonas*, and *Enterobacter*, the most common being the former two. However, when retrospectively analysed, none of these organisms was associated with a successful catheter drain outcome, nor did they predict a failure. Our study found that all patients with *Acinetobacter *isolate in the drain had a poor prognosis, failed percutaneous catheter drainage, or death. Nevertheless, this was not statistically significant.

Limitations

There were a few limitations to our study. It was a single-centre trial with a small sample population size because of the time limitations. The univariate analysis needed a minimum of five subjects in each subset, failing which the statistical analysis may not be proved significantly. This happened in a few variables in our study like female gender, single organ failure, CTSI scores, etc. Association factors for mortality have not been studied.

## Conclusions

In acute necrotising pancreatitis complicated by infective collections, patients with no organ involvement respond better to primary catheter drainage. This modality can be considered the first option in draining the necrotic collections before adopting a more invasive procedure. Patients with BMI>25, multi-organ failure, and a BISAP score of ≥4 are negative predictors for primary catheter drainage of pancreatic necrosis. Further studies are required to explore these predictive factors in a larger sample size to predict the success of catheter drainage in infected pancreatic necrosis.
